# Influences of Carrion Decomposition on Soil Nutrient Leakage in a Boreal Forest

**DOI:** 10.1002/ece3.73973

**Published:** 2026-07-05

**Authors:** E. Wenting, M. P. Arnberg, D. F. Delsman, H. Siepel, F. van Langevelde, S. M. J. G. Steyaert

**Affiliations:** ^1^ Department of Ecology Radboud University, Radboud Institute for Biological and Environmental Sciences Nijmegen the Netherlands; ^2^ Biodiversity Research Institute (CSIC—University of Oviedo—Principality of Asturias) Mieres Spain; ^3^ Faculty of Biosciences and Aquaculture, Terrestrial Ecology Group Nord University Steinkjer Norway; ^4^ Department of Environmental Sciences Wageningen University and Research Wageningen the Netherlands

**Keywords:** biochemistry, boreal forest, carrion ecology, decomposition, trace elements, zoogeochemistry

## Abstract

Carrion decomposition can significantly alter local soil chemical composition. Although a wide range of chemical elements is essential for plants and/or animals, hence ecosystem functioning, most studies on biochemical effects of carrion decomposition have focused on only a few macronutrients. Moreover, existing studies have been conducted in temperate and (semi‐)arid ecosystems, leaving a knowledge gap for biochemical effects in boreal ecosystems, where carrion decomposition is generally slower. Here, we examined how carrion decomposition time, estimated based on vertebrate scavenger activity, influenced 25 soil elemental concentrations and soil pH at carrion sites in a boreal forest in Trøndelag, Norway. We found that longer estimated decomposition times resulted in increased concentrations of nitrogen (N), sodium (Na) and boron (B) in the soil. Some of these elements may be limiting primary production and are likely to be scarce in the study area (e.g., N and B). We encourage further investigation of the consequences of carrion decomposition on local soil biochemistry across ecosystems, taking local abiotic and biotic conditions into account.

## Graphical abstract

We studied elemental leakage into the soil due to carrion decomposition in a boreal forest in Norway. We found that longer carrion decomposition times resulted in increased concentrations of N, Na and B in the soil. To our knowledge, we are the first that included a wide range of chemical elements in our analysis, including macro and trace elements beyond the Redfield ratio (C, N, P).

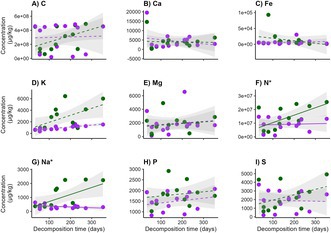

## 1 Introduction

Carrion—i.e., dead animal matter—acts as a pulse resource that can severely alter local soil chemical composition (e.g., Barton et al. 2019; Quaggiotto et al. 2019; Wenting, Jansen, et al. 2023; Wenting, Jansen, Burggraeve, et al. 2024). Its decomposition can result in fertile hotspots through time and space (e.g., Carter et al. 2007), with potentially long‐lasting effects on local soil biochemistry (e.g., Towne 2000; Yang et al. 2022; Monk et al. 2024). Most studies concerning effects of carrion decomposition focused on temperate (e.g., Melis et al. 2007; Macdonald et al. 2014; Wenting, Jansen, Burggraeve, et al. 2024) or (semi‐)arid (e.g., Parmenter and MacMahon 2009; Hu et al. 2023) ecosystems. Although decomposition processes have been studied in colder regions (e.g., Meyer et al. 2013; Wheeler et al. 2014; Wheeler and Kavanagh 2017; Pecsi et al. 2025), studies focusing on carrion‐driven changes in soil biochemistry across a broad range of elements in boreal ecosystems remain limited. This represents a major knowledge gap, given that nutrient cycling and carrion decomposition generally proceed slow in these ecosystems.

Moreover, most studies across all ecosystems have focused on a limited number of macronutrients released from carrion, such as carbon (C), nitrogen (N) and phosphorus (P) (e.g., Bump et al. 2009; Parmenter and MacMahon 2009; Keenan et al. 2019), despite many other elements being essential for plants and/or animals (Robinson et al. 2009; Kaspari 2021), and thus ecosystem functioning. Several studies have also examined additional macro elements, including Ca, Mg, K, Na and S, which can be released during decomposition of soft tissues, blood and bone material (e.g., Parmenter and MacMahon 2009; Perrault and Forbes 2016; Keenan and Beeler 2023). Some recent studies have expanded this scope by measuring a wider range of elements, including trace elements. For example, Taylor et al. (2023) measured 14 elements over time and found long‐lasting changes in local soil nutrient pools in a temperate mixed deciduous forest, while Wenting, Jansen, Burggraeve, et al. (2024) measured 22 elements during carrion decomposition in a temperate forest and found that potentially scarce trace elements showed the largest response, in concentrations that can enhance net plant growth (Wenting, Jansen, et al. 2023). However, how carrion decomposition influences a wide range of elements in boreal forests remains unknown.

Carrion decomposition and associated nutrient leakage are strongly context dependent. Elemental limitation varies among ecosystems and may be shaped by local factors such as anthropogenic pollution and atmospheric nitrogen deposition (e.g., Siepel et al. 2018, 2019; Barjoee et al. 2021; Rubin et al. 2023). Scavenger activity is a key biotic driver of this context dependence: vertebrate scavengers can substantially shorten carrion decomposition time (e.g., Peers et al. 2021; Newsome et al. 2021; Wenting, Jansen, Pattipeilohy, et al. 2024; Wenting, Siepel, et al. 2024) and reduce elemental leakage into the soil (Wenting, Jansen, Burggraeve, et al. 2024). In contrast, slower decomposition dominated by invertebrates and microbes can result in locally elevated concentrations of potentially scarce elements, particularly copper (Cu) and zinc (Zn). Consequently, carrion decomposition may influence soil biochemistry differently across geographical regions and ecosystems.

The objective of this study was to assess how estimated duration of carrion decomposition influenced local soil biochemistry, including a wide range of elements and soil pH, in a boreal forest. We expected changes for the scarcest elements in the study area, which are present in animal biomass in relatively high concentrations (e.g., Ma et al. 2015; Wenting et al. 2020; Wenting, Siepel, and Jansen 2023), that we used as carrion. Leakages of such elements that are highly accumulated in animal bodies compared to background concentrations in the soil, are predicted to have a stronger effect on local soil biochemistry due to carrion decomposition, as demonstrated in previous studies (e.g., Wenting, Jansen, et al. 2023; Wenting, Jansen, Burggraeve, et al. 2024). Given the identified knowledge gaps and potential area‐dependent results, we did not formulate explicit predictions per element. We performed a field experiment, in which we experimentally placed carrion in the municipality of Steinkjer, county Trøndelag, Norway, estimated the carrion decomposition time based on vertebrate scavenger activity, and measured elemental concentrations and soil pH following decomposition.

## 2 Material and Methods

**FIGURE 1:**
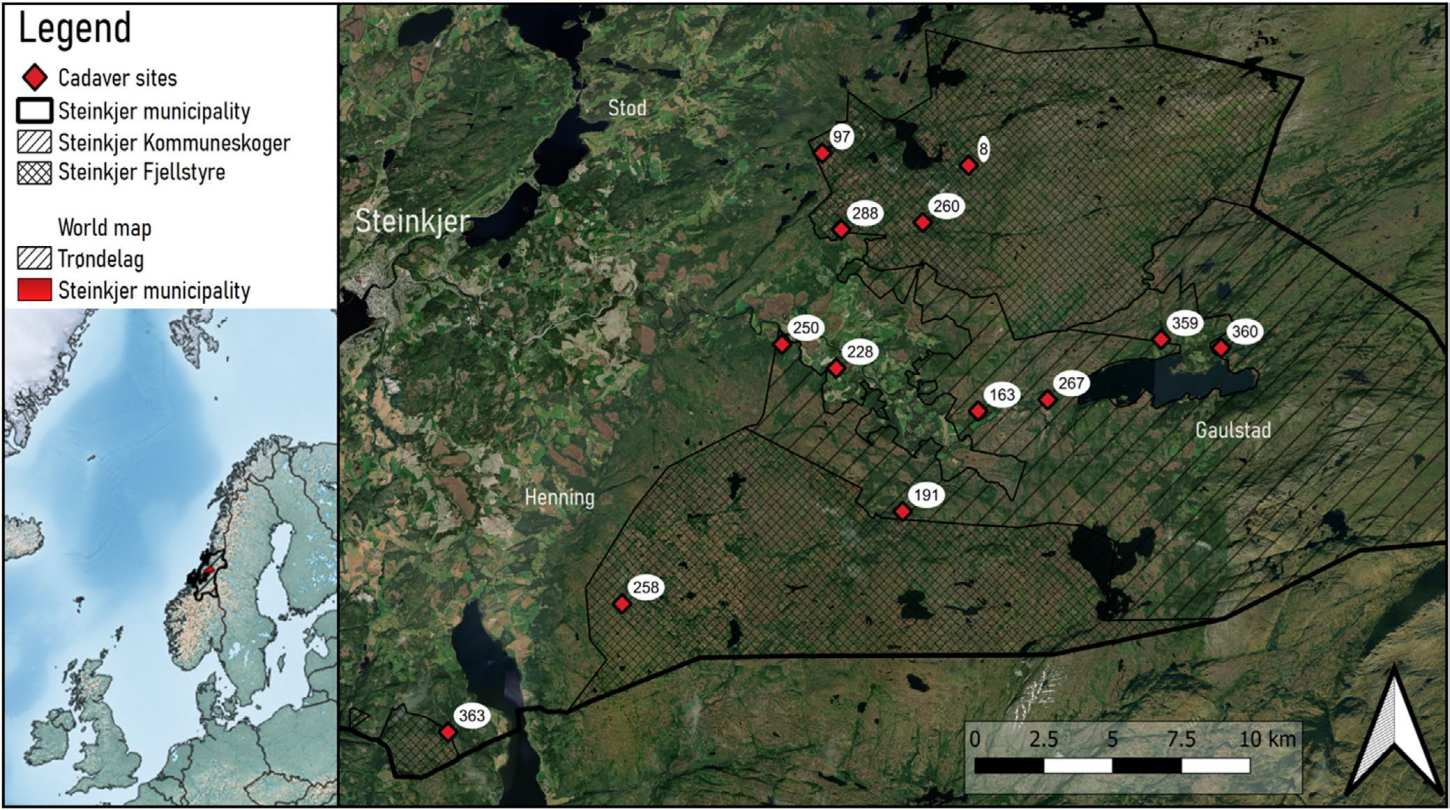
Overview of the carrion deployment sites, each consisting of a carrion plot and a paired control plot, located within the administrative borders of Steinkjer Kommuneskoger and Steinkjer Fjellstyre in county Trøndelag, Norway. Numbers indicates site location. The red dots represent the carrion deployments included in this study. See Table 1 for details about the deployments.

This study was part of a larger experiment investigating the effect of carrion decomposition on vegetation and seedling establishment of berry‐producing ericaceous species (Arnberg et al. 2024). The experiment was conducted within the administrative borders of Steinkjer Kommuneskoger (municipal forest service) and Steinkjer Fjellstyre (administrative body tasked with, among others, overseeing hunting and forestry), situated in the municipality of Steinkjer, Trøndelag county, Norway (Figure 1; 64°01’N, 11°42′ E).

The study area has a boreal climate (Dfc; Köppen classification; Peel et al. 2007), annual precipitation of approximately 1069 mm, and mean annual air temperatures ranging from 4.3°C to 5.7°C (based on a five‐year period from the weather stations Snåsa SN70850 and Verdal SN70510; The Norwegian Meteorological Institute; MET Norway 2022). The soils include peat and podzolic soils on which bilberry spruce forests are dominantly present (Reimann et al. 2015; Sørensen et al. 2022).

**FIGURE 2:**
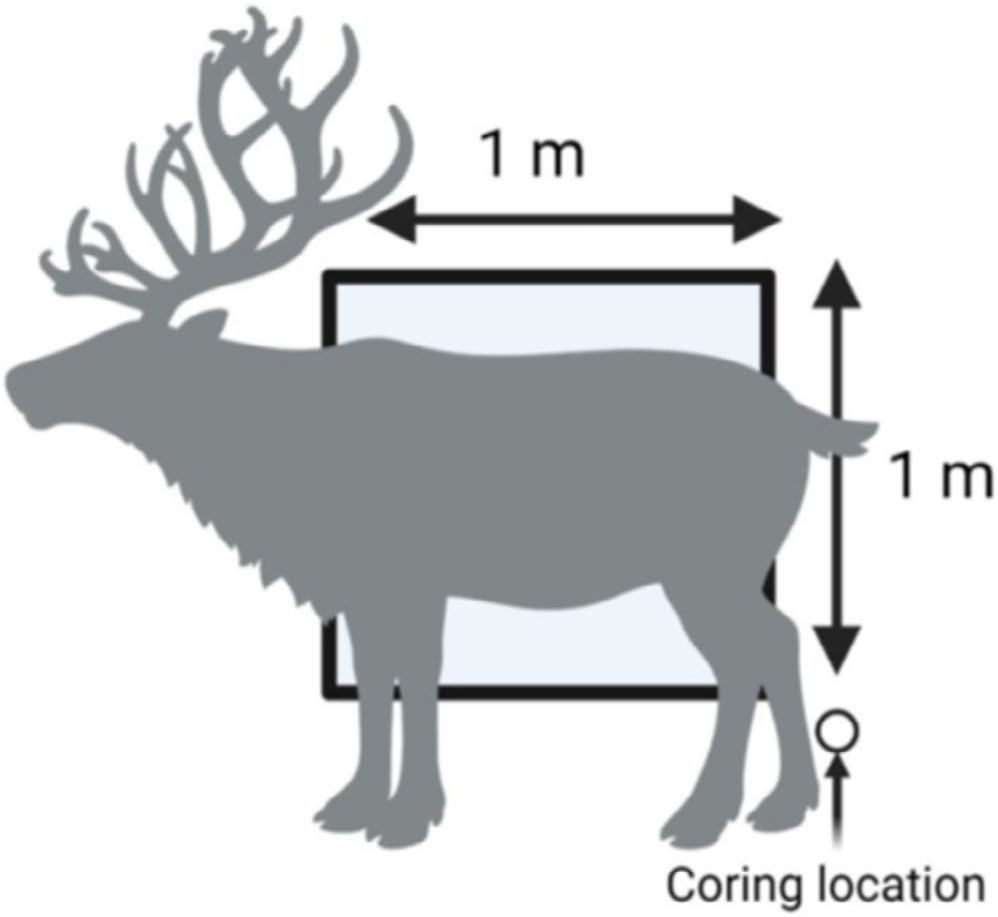
Schematic overview of the carrion placement and soil sampling location.

Carrion deployment sites were randomly selected and met four criteria: (1) located in forest; (2) 100 to 1000 m from any roads; (3) no buildings within 100 m; and (4) slope ≤ 20° (Arnberg et al. 2024). Each site consisted of a carrion plot and a paired control plot, located 30–50 m apart within the same habitat type. At each plot, a grid of 1 × 1 m was established and carrion was placed centrally on its flank in carrion plots (Figure 2) in August or September 2020. Carrion originated from hunting remains or roadkill, based on availability. No animals were killed for the purpose of this study. We used 13 carrion deployments for which decomposition time could be estimated: one cow ( *Bos taurus* ; Linnaeus 1758), nine moose ( *Alces alces* ; Linnaeus 1758) and three reindeer ( *Rangifer tarandus* ; Linnaeus 1758). All carrion deployments started in summer 2020 and soil samples were taken at the start of the deployment and in spring/summer 2021 (Table 1). Decomposition time was approximated as the period between carrion placement and the cessation of vertebrate scavenger activity (i.e., the point in time that the carrion did no longer attract scavengers), monitored using camera traps (see Arnberg et al. 2024 for details). We used this estimation of decomposition time because sampling date alone did not capture variation in the duration of carrion influence prior to sampling, and estimated decomposition time was therefore used as a site‐level covariate. Soil samples—5 cm top soil, dried at 40°C for 48 h and sieved before further processing—were taken before and approximately 1 year after the start of the carrion deployment, between March and September 2021 (Table 1), at the edge of the south‐eastern corner of the grids with a soil core sampler (Figure 2). Due to its size, the carrion covered the entire 1 × 1 m plot and beyond, i.e., the samples were taken on spots where carrion had decomposed but not at the centre of the carrion. The difference between the estimated decomposition time and the moment the soil sampling was used as a covariate in the statistical analyses.

**TABLE 1:** Overview of the carrion deployments used in this study.

Deployment site	Type of carrion	Start date deployment	Estimated decomposition time (EDT) (days)	Difference between EDT and sampling date (days)
8	Moose	02/09/2020	222	108
97	Moose	02/09/2020	353	37
163	Moose	31/08/2020	56	274
191	Reindeer	07/09/2020	57	183
228	Reindeer	14/09/2020	34	206
250	Moose	02/09/2020	110	220
258	Moose	30/08/2020	272	58
260	Moose	11/09/2020	211	119
267	Moose	01/09/2020	234	96
288	Moose	11/09/2020	79	251
359	Reindeer	21/08/2020	136	74
360	Cow	21/08/2020	131	109
363	Moose	14/09/2020	175	65

The soil samples were transported to The Netherlands for further analyses. At Wageningen University and Research, we measured the soil pH using an Inolab pH‐metre (calibrated at pH 7 and 4) and, after the samples were ground and placed in tin capsuled, we measured C and N using a LECO CN 628 Dumas analyser (combustion procedure). At Radboud University, we used a microwave destruction—aka digestion—method with 5 mL 65% nitric acid (HNO_3_) and 2 mL 30% hydrogen peroxide (H_2_O_2_) prior to measuring 23 elemental concentrations. This included seven other macro elements (Robinson et al. 2009; Kaspari 2021): calcium (Ca), iron (Fe), potassium (K), magnesium (Mg), sodium (Na), P, and sulphur (S). Another 12 elements were classified as trace elements (Robinson et al. 2009; Kaspari 2021): boron (B), barium (Ba), cobalt (Co), chromium (Cr), copper (Cu), manganese (Mn), molybdenum (Mo), nickel (Ni), selenium (Se), silicon (Si), strontium (Sr) and zinc (Zn). The remaining four elements—i.e., aluminium (Al), arsenic (As), cadmium (Cd) and lead (Pb)—were classified as potentially ecotoxic elements that have no function for plants or animals (Robinson et al. 2009; Kaspari 2021; Wenting, Siepel, and Jansen 2023). Eight elements (Al, Ca, K, Mg, Na, P, S, Si) were measured using Inductively Coupled Plasma Optical Emission Spectrometry (ICP‐OES), while the other elements were analysed using Inductively Coupled Plasma Mass Spectrometry (ICP‐MS).

Statistical analyses were done in R version 4.4.1 (R Core Team 2024) using linear mixed‐effects models with the “lmer” function of the “lmerTest” package (Kuznetsova et al. 2017). Elemental concentration and soil pH were used as response variables, with the difference between estimated decomposition time and sampling date, and interaction between estimated decomposition time and sample type (i.e., carrion deployment or control) as fixed effects. Deployment location and species of carrion were included as random factors (see Appendix S1 for all test statistics, Table S1–S8). Model diagnostics indicated no violations of model assumptions (Appendix S1, Figure S1).

We added the interaction effect between estimated decomposition time and sample type to our models, as this captures different trends between the two plot types. Interpretation of models was based primarily on the statistical significance of the interaction between decomposition time and sample type. Due to the limited sample size, visual inspection of figures was used only for descriptive support and not for inference. In addition, we compared the initial conditions between the carrion and control plot as well as before and after carrion deployment within both plots, using *t*‐tests.

No data were available on the elemental composition of the carrion sources (i.e., the ionome; e.g., Lahner et al. 2003; Salt et al. 2008; Ma et al. 2012; Wenting et al. 2020; Wenting, Siepel, and Jansen 2023). Because mammalian ionomes are largely species‐specific (e.g., Prater et al. 2019; Wenting et al. 2020; Van Beest et al. 2024) and may vary among geographical regions (e.g., Oropesa et al. 2022; Wenting, Siepel, and Jansen 2023; Van Beest et al. 2024), we did not formulate element‐specific predictions prior to the analyses.

## 3 Results

### 3.1 Macro Elements

**FIGURE 3:**
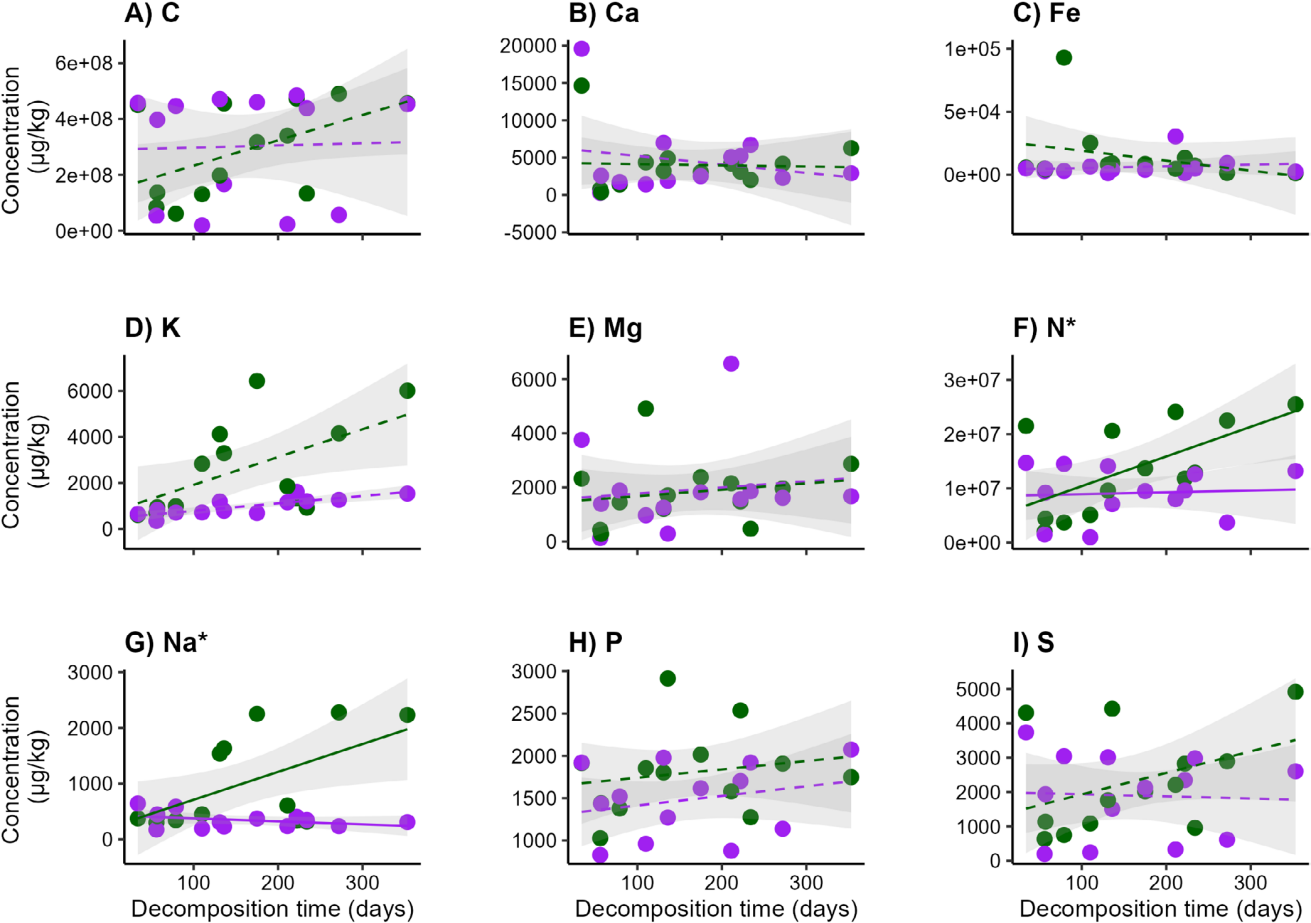
Concentrations of macro elements in soil after carrion deployment based on the estimated decomposition time. Green dots represent the carrion sites and purple dots represent the control sites. Asterisks (*) indicates statistically significant effects (*p* < 0.05). Dashed lines represent non‐significant trends.

Initial elemental concentrations of macro elements did not differ between the carrion and control plot (Appendix S1). However, C concentration increased after carrion deployment in the carrion plot (*t* = −3.78, df = 34.0, *p* = 0.016), as well as K concentration increasing in the control plot (*t* = 4.89, df = 37.87, *p* < 0.001). Longer decomposition times were associated with significantly higher soil concentrations of N and Na (Figure 3F,G; Table 2). K and S showed increasing trends with decomposition time, although not significant (Figure 3D,I; Table 2). No other macro elements showed significant or visually apparent trends.

**TABLE 2:** Test statistics of the interaction effect between estimated decomposition time and sample type (carrion or control site).

Element	Type	NumDF	DenDF	*F*	*p*
C	Macro element	1	5.59E+20	1.257	0.262
Ca	Macro element	1	1.32E+25	1.252	0.263
Fe	Macro element	1	19.92712	2.277	0.147
K	Macro element	1	2.11E+40	3.041	0.081
Mg	Macro element	1	2.02E+09	0.000	0.985
N	Macro element	1	218.7602	7.495	0.007*
Na	Macro element	1	2.26E+35	7.408	0.006*
P	Macro element	1	1.45E+37	0.006	0.938
S	Macro element	1	287.3103	4.245	0.040*
B	Trace element	1	11	6.208	0.030*
Ba	Trace element	1	21	0.000	0.986
Co	Trace element	1	18.57881	1.181	0.291
Cr	Trace element	1	11.73475	0.166	0.691
Cu	Trace element	1	9.884653	2.944	0.117
Mn	Trace element	1	20.16941	1.294	0.269
Mo	Trace element	1	10.69797	1.078	0.322
Ni	Trace element	1	21	0.124	0.728
Se	Trace element	1	20.77253	10.258	0.004*
Si	Trace element	1	105.9889	5.241	0.024*
Sr	Trace element	1	11	1.367	0.267
Zn	Trace element	1	21	3.037	0.096
Al	Ecotoxic element	1	252.1581	4.388	0.037*
As	Ecotoxic element	1	19.72135	2.340	0.142
Cd	Ecotoxic element	1	21	1.369	0.255
Pb	Ecotoxic element	1	10.99999	0.133	0.722
pH	pH	1	15.3223	0.191	0.668

*Note:* Asterisks (*) indicate statistically significant effects (*p* < 0.05).

### 3.2 Trace Elements

**FIGURE 4:**
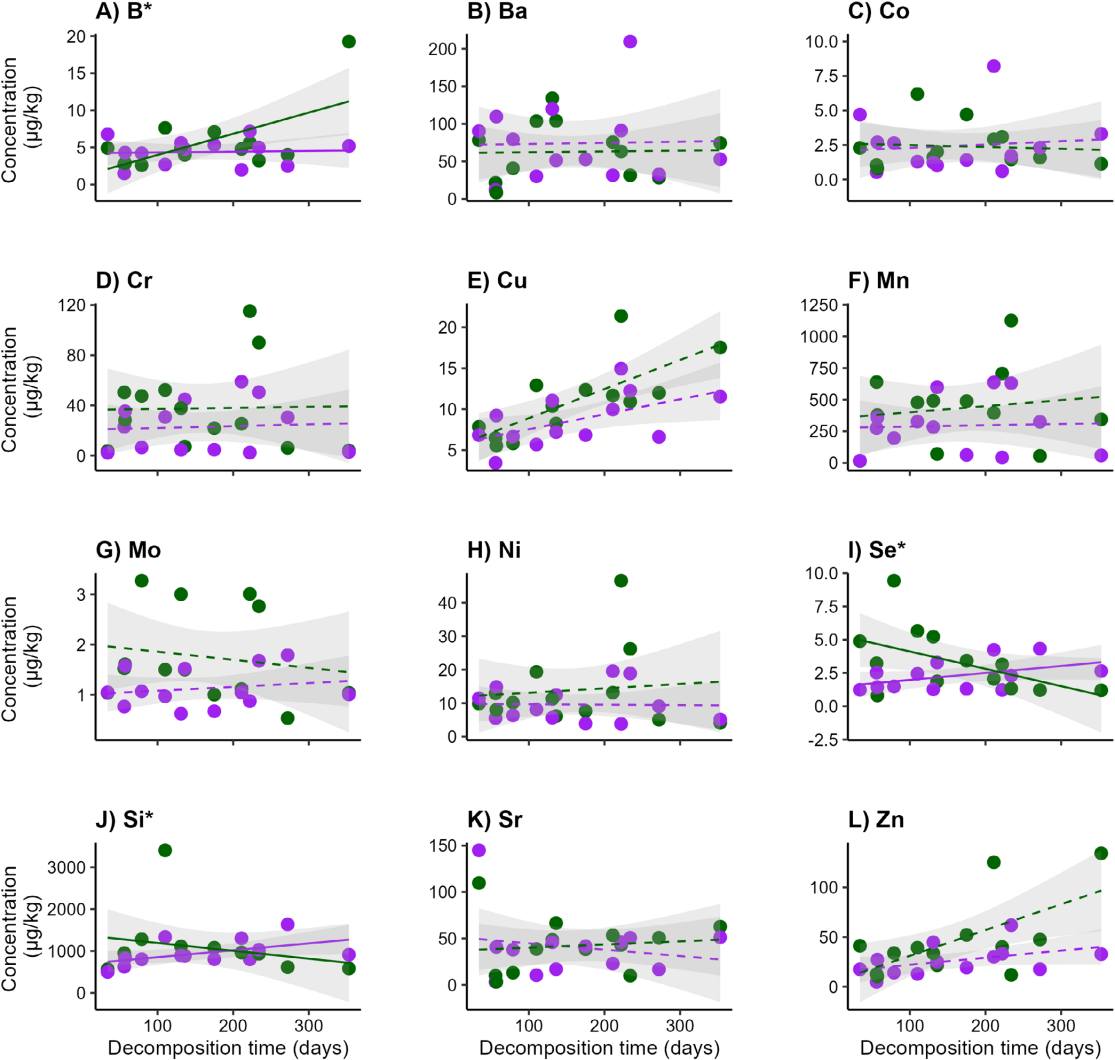
Concentrations of trace elements in soil after carrion deployment based on the estimated decomposition time. Green dots represent the carrion sites and purple dots represent the control sites. Asterisks (*) indicates statistically significant effects (*p* < 0.05). Dashed lines represent non‐significant trends.

For the trace elements, no differences were detected between carrion and control plot in initial element concentration, or before versus after carrion deployment (Appendix S1). B concentrations increased significantly with longer decomposition times (Figure 4A; Table 2). The trends of Se and Si were also statistically significant (Figure 4I,J; Table 2), although no clear directional trends were evident relative to the control. Cu and Zn showed visibly increasing trends for both carrion and control plots with decomposition time, but no significant interaction effect was detected (Figure 4E,L; Table 2).

### 3.3 (Eco)toxic Elements

**FIGURE 5:**
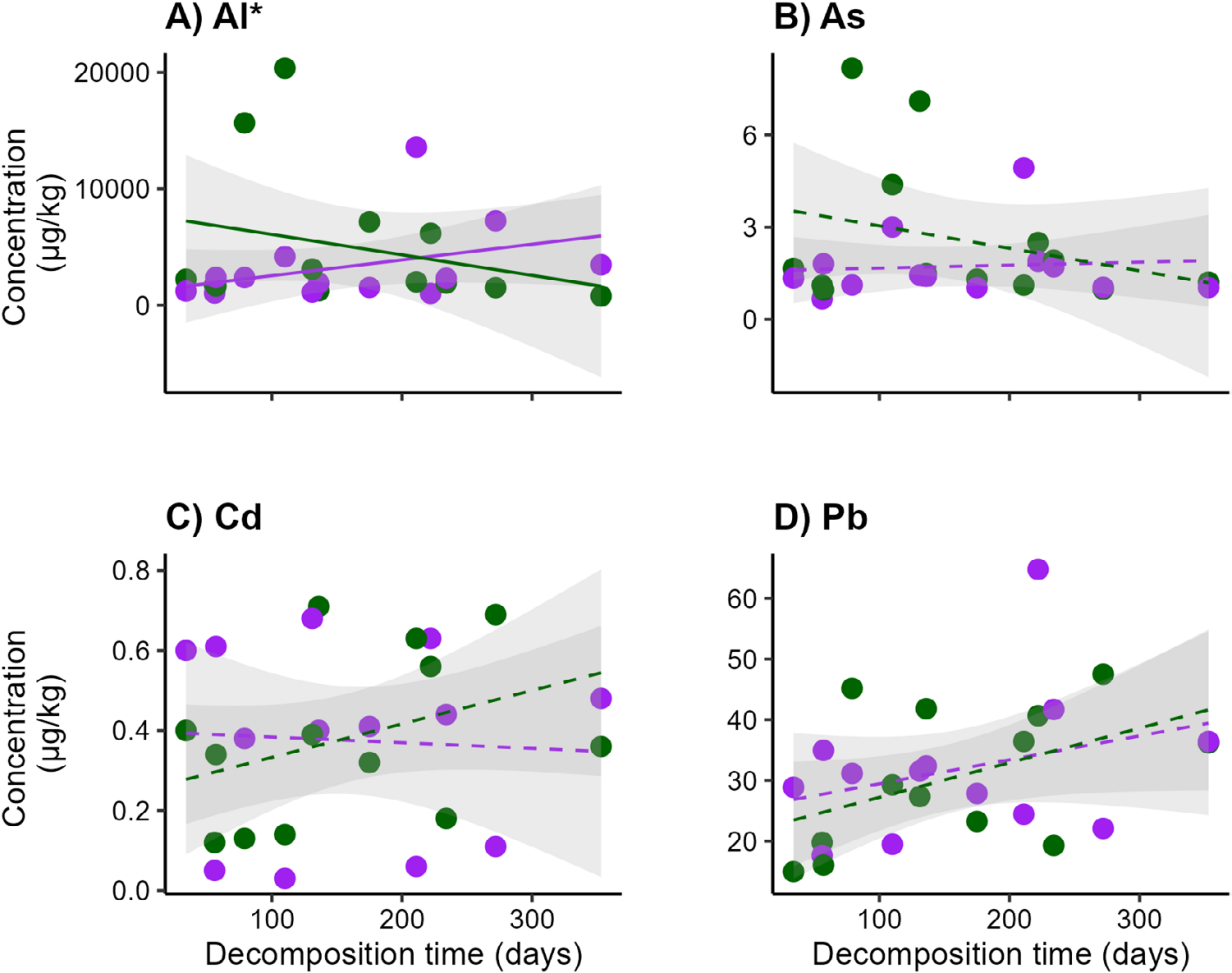
Concentrations of potentially (eco)toxic elements in soil after carrion deployment based on the estimated decomposition time. Green dots represent the carrion sites and purple dots represent the control sites. Asterisks (*) indicates statistically significant effects (*p* < 0.05). Dashed lines represent non‐significant trends.

No differences were found between initial values of the carrion and control plot, or between sampling rounds (Appendix S1). Al showed a statistically significant interaction effect (Table 2), with opposing directional changes between the carrion and control plots (Figure 5A). Concentrations increased with decomposition time in the carrion plots, while decreasing in control plots. However, the apparent magnitude of change is influenced by the scale and variability was high, and therefore the pattern should be interpreted with caution. We did not observe any trend that we could clearly relate to carrion decomposition time for any of the other potentially (eco) toxic elements (Figure 5).

### 3.4 Soil pH

**FIGURE 6:**
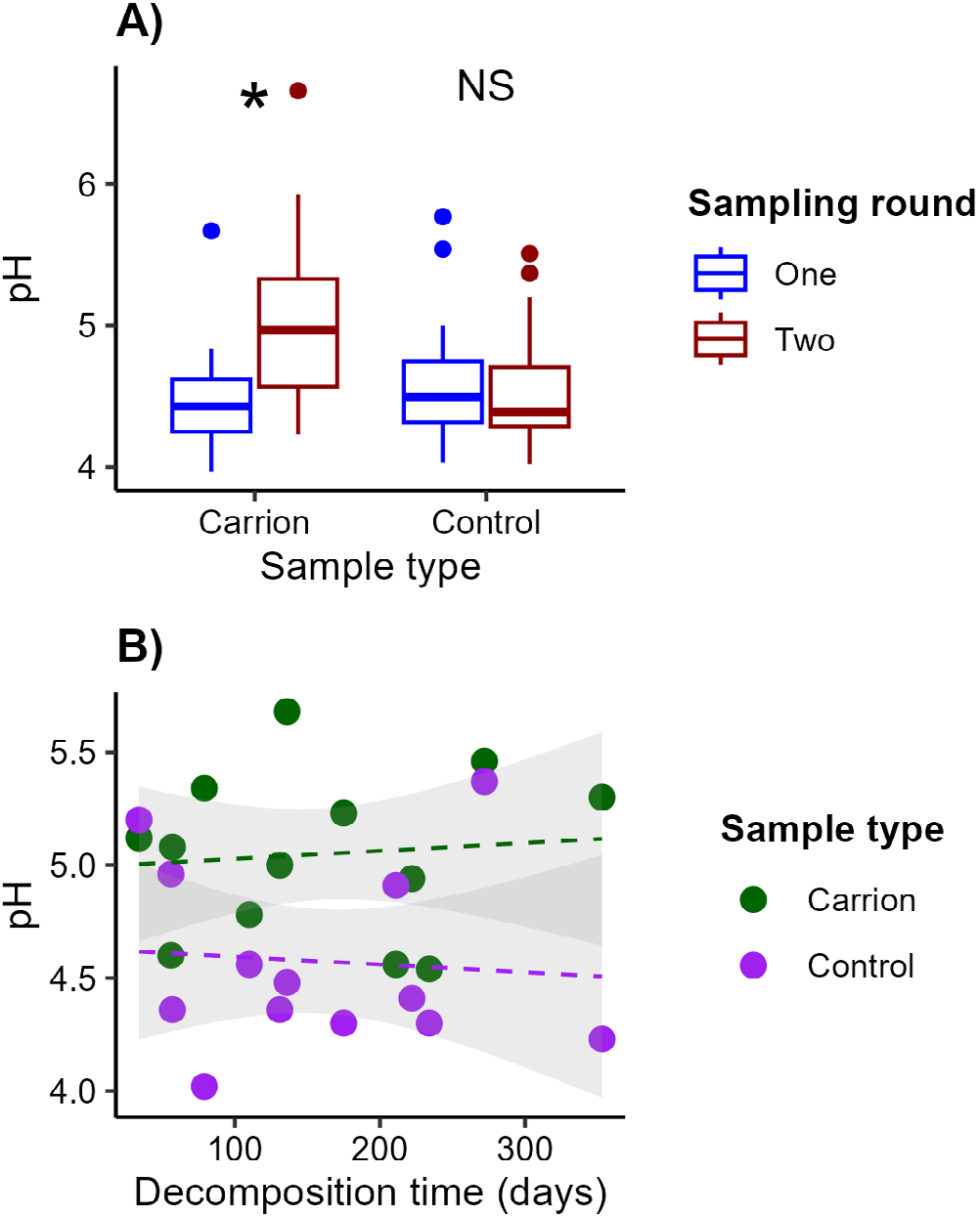
Changes in soil pH after carrion deployment (A) and over time based on the estimated decomposition time (B). Green dots represent the carrion sites and purple dots represent the control sites. *p < 0.05. Dashed lines represent non‐significant trends.

Soil pH did not differ between the carrion and control plot before carrion placement (*t*‐test, *t* = 0.991, df = 43.5, *p* = 0.849). The soil pH increased on the carrion plot after the decomposition (Figure 6A; *t*‐test, *t* = −3.780, df = 34.0, *p* = 0.016) but not on the control plot (Figure 6A; *t*‐test, df = 0.406, df = 45.7, *p* = 0.986). Changes in soil pH were not related to decomposition time (Figure 6B; Table 2).

## 4 Discussion

### 4.1 Macro Elements

The increased soil concentrations of N and Na with longer decomposition times are consistent with previous carrion decomposition studies (e.g., Parmenter and MacMahon 2009; Spicka et al. 2011; Keenan et al. 2018, 2019). Carrion‐derived nutrient inputs can persist for varying durations, with carrion acting as an important source of N particularly during the first year after decomposition in warmer climates (Melis et al. 2007; Macdonald et al. 2014), but for up to 5 years in colder ecosystems with lower nutrient turnover rates (Danell et al. 2002). Carrion is generally rich in N (Carter et al. 2007), and its decomposition can therefore cause substantial increases in local soil N pools, especially in nutrient‐poor and relatively cold ecosystems such as boreal forests (Gower et al. 2000). Similarly, C concentrations can persists for multiple years and may follow non‐linear temporal patterns (Singh et al. 2018), which may exceed the timeframe of our study (Singh et al. 2018).

Although Na is not classified as an essential nutrient for most plants, it can play an important role in plant physiology under certain conditions (e.g., Subbarao et al. 2003; Kaspari 2020), it is readily taken up when available and is essential for animals (Subbarao et al. 2003; Doughty et al. 2016; Kaspari 2021). Long‐lasting increases in soil Na following carrion decomposition have been reported (Aitkenhead‐Peterson et al. 2012; Fancher et al. 2017), but plant responses remain poorly understood (Perrault and Forbes 2016). Na‐enriched vegetation may influence foraging behaviour of herbivore (Villalba et al. 2008; DeSimone et al. 2013; Duvall et al. 2023), suggesting that carrion‐derived Na could indirectly influence other ecosystem processes, although selective feeding by animals for specific elements remains poorly studied (Pringle et al. 2023).

We observed non‐significant increasing trends for K and S, which aligns with other studies reporting elevated K during decomposition (Parmenter and MacMahon 2009; Fancher et al. 2017; Szelecz et al. 2018). In contrast, no K increase was found in systems heavily affected by anthropogenic N deposition and long‐lasting acidification (Wenting, Jansen, Burggraeve, et al. 2024), suggesting that background nutrient imbalances may directly influence the effects of carrion decomposition on soils. S release during decomposition is primarily associated with soft tissues and volatile compounds responsible for the typical “corpse smell,” which attracts invertebrates such as blowflies (Parmenter and MacMahon 2009; Verheggen et al. 2017), potentially accelerating decomposition and influencing vertebrate scavenger dynamics (e.g., Pechal et al. 2014; Olson et al. 2022). Contrasting to previous studies (e.g., Towne 2000; Macdonald et al. 2014; Quaggiotto et al. 2019), we did not find increased P concentration relative to decomposition time. The underlying causes remain unclear based on our data, highlighting the need for further investigation in boreal systems.

### 4.2 Trace Elements

B concentrations increased significantly with longer decomposition times, consistent with findings from temperate forests (Taylor et al. 2023). B is essential for plants and animals and is prone to leaching in regions with high‐precipitation (Saleem et al. 2011; Pizzorno 2015). There is an increasing number of studies suggesting that B may be among the scarcest trace elements in Norway (e.g., Kilpeläinen et al. 2013; Vartiainen et al. 2025). Our result could therefore support our prediction that carrion decomposition disproportionately affects locally limiting elements.

Although not statistically significant, increasing trends for Cu and Zn were observed consistent with previous studies showing carrion‐induced enrichment of these elements (Wenting, Jansen, et al. 2023; Wenting, Jansen, Burggraeve, et al. 2024). Zn, in particular, is globally scarce in many soils (Moreno‐Jiménez et al. 2019), which may explain its sensitivity to carrion‐derived inputs. In contrast, Se and Si concentrations decreased, although these patterns were not consistently different between the carrion and control plots and therefore could not be clearly attributed to carrion‐specific effects. This may reflect shifts in chemical availability during decomposition that cannot be explained by our data, therefore indicating that carrion decomposition does not universally lead to increased elemental concentrations.

### 4.3 Potentially (Eco)toxic Elements

We did not detect consistent carrion‐specific patterns for most of the measured potentially (eco)toxic elements. Al showed a significant interaction effect, with opposing trends between carrion and control plots. However, variability was high and the underlying mechanisms remain unclear. Therefore, we refrain from further interpretation.

### 4.4 Soil pH

Soil pH increased on carrion plots relative to controls but was not related to the decomposition time. Previous studies have reported highly variable pH responses to carrion decomposition (e.g., Keenan et al. 2019; Quaggiotto et al. 2019; Barton et al. 2020), indicating strong context dependence. The peat and podzol soils may exhibit complex chemical behaviour (Muir 1961; Walmsley 1977), which may have influenced the observed patterns.

### 4.5 Limitations

A major limitation in our sampling design is the location of soil coring. Although the carrion covered the entire 1 × 1 m plot size at placement, this was likely not the case throughout the entire decomposition process. Previous studies found little to no lateral spread of carrion‐derived elements beyond approximately 30 cm from carrion (e.g., Melis et al. 2007; Keenan et al. 2019; Barton et al. 2020), and such lateral spread may be non‐uniform or asymmetrical (Aitkenhead‐Peterson et al. 2012). This limited lateral spread may explain why we primarily detected increased concentrations of relatively mobile elements in soils such as B (e.g., Tanasheva et al. 2012; Das and Purkait 2020). Moreover, we acknowledge that, although carrion and control plots were paired within the same habitat and showed no significant differences in initial soil elemental concentrations, small‐scale spatial heterogeneity in soil properties may still have influenced observed variation. Given the 30–50 m distance between paired plots, we cannot fully exclude that part of the observed differences is driven by natural spatial variability rather than carrion‐derived inputs. For future studies, we recommend collecting soil samples directly beneath or within close proximity (< 20 cm) to the centre of decomposing carrion. The limited sample size constrains the strength of our inference. However, given the scarcity of multi‐element carrion studies (Taylor et al. 2023; Wenting, Jansen, Burggraeve, et al. 2024), especially in boreal regions, our findings provide valuable insights into how local conditions may shape carrion‐driven soil biochemistry.

In this study, carrion decomposition time was estimated based on the activity of vertebrate scavengers and should therefore be interpreted with caution. Carrion decomposition and scavenger activity are inherently variable and influenced by multiple biotic and abiotic factors (e.g., Inagaki et al. 2020), including temperature, moisture, landscape characteristics and seasonal variations (e.g., Turner et al. 2017; Barton et al. 2019; Oliva‐Vidal et al. 2022; Englmeier et al. 2023; Wenting, Jansen, Pattipeilohy, et al. 2024). In boreal forests, snow and frost may conceal carcasses for extended periods, delaying detection by vertebrate scavengers and altering decomposition dynamics. Longer decomposition times increase the likelihood of scavenger detection, but not necessarily increased consumption (Mateo‐Tomás et al. 2019; Wenting et al. 2022). Slower carrion decomposition is more likely to be dominated by invertebrate scavengers and microbial decomposers (Janzen 1977), reducing vertebrate scavenger activity (Forbes et al. 2022; Séguin et al. 2022). However, our annual soil sampling schedule and limited sample size prevents assessment of seasonal or microbial effects (Burcham et al. 2024). Nevertheless, carrion decomposition dominated by invertebrates and microbes has been demonstrated to result in greater elemental leakage into the soil than decomposition dominated by vertebrate scavengers (Wenting, Jansen, Burggraeve, et al. 2024).

## 5 Conclusions

In conclusion, our study demonstrates that carrion decomposition has localised effects on soil biochemistry in boreal forests. Longer decomposition times resulted in greater elemental leakage of potentially scarce chemical elements, including elements that potentially limit plant growth, such as N (Figure 3F) and B (Figure 4A). We encourage further investigation of carrion‐driven soil biochemistry across ecosystems, including boreal forests, with explicit consideration of local abiotic (e.g., nutrient availability, precipitation, snow cover duration) and biotic (e.g., scavenger activity) conditions.

## Author Contributions

**E. Wenting:** conceptualization (supporting), formal analysis (lead), investigation (supporting), visualization (lead), writing – original draft (lead), writing – review and editing (equal). **M. P. Arnberg:** conceptualization (equal), investigation (equal), methodology (equal), project administration (lead), writing – original draft (supporting), writing – review and editing (equal). **D. F. Delsman:** investigation (equal), writing – review and editing (equal). **H. Siepel:** formal analysis (supporting), resources (equal), visualization (supporting), writing – review and editing (equal). **F. van Langevelde:** conceptualization (supporting), resources (equal), writing – review and editing (equal). **S. M. J. G. Steyaert:** conceptualization (equal), methodology (equal), resources (equal), writing – review and editing (equal).

## Funding

This research was funded through a research development grant (Grant number 30028‐111) (Nord University).

## Conflicts of Interest

The authors declare no conflicts of interest.

## Supporting information


**Table S1:** All test statistics belonging to the macro elements; comparison carrion and control plot before and after carrion deployment (Welch Two Sample *t*‐test). The column “Adjusted *p*” reports the *p‐*values after correction for multiple testing based on Benjamini & Hochberg (1995).
**Table S2:** All test statistics belonging to the macro elements; elemental concentrations related to estimated decomposition time. DT = estimated decomposition time; ST = sample type (carrion or control plot); DD = difference between estimated decomposition time and sampling date; INTER = interaction DT and ST.
**Table S3:** All test statistics belonging to the trace elements; comparison carrion and control plot before and after carrion deployment (Welch Two Sample *t*‐test). The column “Adjusted *p*” reports the *p‐*values after correction for multiple testing based on Benjamini & Hochberg (1995).
**Table S4:** All test statistics belonging to the trace elements; elemental concentrations related to estimated decomposition time. DT = estimated decomposition time; ST = sample type (carrion or control plot); DD = difference between estimated decomposition time and sampling date; INTER = interaction DT and ST.
**Table S5:** All test statistics belonging to the ecotoxic elements; comparison carrion and control plot before and after carrion deployment (Welch Two Sample *t*‐test). The column “Adjusted *p*” reports the *p‐*values after correction for multiple testing based on Benjamini & Hochberg (1995).
**Table S6:** All test statistics belonging to the ecotoxic elements; elemental concentrations related to estimated decomposition time. DT = estimated decomposition time; ST = sample type (carrion or control plot); DD = difference between estimated decomposition time and sampling date; INTER = interaction DT and ST.
**Table S7:** All test statistics belonging to the soil pH; comparison carrion and control plot before and after carrion deployment (Welch Two Sample *t*‐test). The column “Adjusted *p*” reports the *p‐*values after correction for multiple testing based on Benjamini & Hochberg (1995).
**Table S8:** All test statistics belonging to the soil pH; elemental concentrations related to estimated decomposition time. DT = estimated decomposition time; ST = sample type (carrion or control plot); DD = difference between estimated decomposition time and sampling date; INTER = interaction DT and ST.
**Figure S1:** Test for leverage based on Cook's distance in the linear mixed‐effects models (Table S2 + 4 + 6 + 8).

## Data Availability

The data used for this manuscript is available via Figshare: https://doi.org/10.6084/m9.figshare.30464759.
