# Supplementary material for: Influences of Carrion Decomposition on Soil Nutrient Leakage in a Boreal Forest
Source: Ecol Evol. 2026 Jul 5;16(7):e73973. doi: 10.1002/ece3.73973 (PMC13333256; doi:10.1002/ece3.73973)
Supplement: Supplementary file 1 — Table S1: All test statistics belonging to the macro elements; comparison carrion and control plot before and after carrion deployment (Welch Two Sample t‐test). The column “Adjusted p” reports the p‐values after correction for multiple testing based on Benjamini & Hochberg (1995). Table S2: All test statistics belonging to the macro elements; elemental concentrations related to estimated decomposition time. DT = estimated decomposition time; ST = sample type (carrion or control plot); DD = difference between estimated decomposition time and sampling date; INTER = interaction DT and ST. Table S3: All test statistics belonging to the trace elements; comparison carrion and control plot before and after carrion deployment (Welch Two Sample t‐test). The column “Adjusted p” reports the p‐values after correction for multiple testing based on Benjamini & Hochberg (1995). Table S4: All test statistics belonging to the trace elements; elemental concentrations related to estimated decomposition time. DT = estimated decomposition time; ST = sample type (carrion or control plot); DD = difference between estimated decomposition time and sampling date; INTER = interaction DT and ST. Table S5: All test statistics belonging to the ecotoxic elements; comparison carrion and control plot before and after carrion deployment (Welch Two Sample t‐test). The column “Adjusted p” reports the p‐values after correction for multiple testing based on Benjamini & Hochberg (1995). Table S6: All test statistics belonging to the ecotoxic elements; elemental concentrations related to estimated decomposition time. DT = estimated decomposition time; ST = sample type (carrion or control plot); DD = difference between estimated decomposition time and sampling date; INTER = interaction DT and ST. Table S7: All test statistics belonging to the soil pH; comparison carrion and control plot before and after carrion deployment (Welch Two Sample t‐test). The column “Adjusted p” reports the p‐values after correc [file ECE3-16-e73973-s001.docx]

**Appendix A – Test statistics belonging to “Influences of carrion decomposition on soil nutrient leakage in a boreal forest”**

Table S1 All test statistics belonging to the macro elements; comparison carrion and control plot before and after carrion deployment (Welch Two Sample t-test). The column “Adjusted p” reports the p values after correction for multiple testing based on Benjamini & Hochberg (1995).

| **Element** | **Comparison** | **t-value** | **DF** | **p value** | **Adjusted p** |
| --- | --- | --- | --- | --- | --- |
| C | Initial concentrations | -0.17788 | 43.707 | 0.8596 | 0.98614286 |
| C | Carrion plot after carrion deployment | -3.7801 | 33.954 | 0.0006061 | 0.0157586 |
| C | Control plot after carrion deployment | 2.2766 | 41.836 | 0.02799 | 0.2729025 |
| Ca | Initial concentrations | -0.88218 | 43.248 | 0.3826 | 0.90432727 |
| Ca | Carrion plot after carrion deployment | 0.43559 | 39.023 | 0.6655 | 0.98614286 |
| Ca | Control plot after carrion deployment | 0.72636 | 38.83 | 0.472 | 0.98614286 |
| Fe | Initial concentrations | -0.041789 | 43.38 | 0.9669 | 0.98614286 |
| Fe | Carrion plot after carrion deployment | -1.1124 | 39.795 | 0.2726 | 0.79415556 |
| Fe | Control plot after carrion deployment | -0.50857 | 45.755 | 0.6135 | 0.98614286 |
| K | Initial concentrations | 0.24526 | 32.613 | 0.8078 | 0.98614286 |
| K | Carrion plot after carrion deployment | -1.8425 | 30.877 | 0.07502 | 0.45012 |
| K | Control plot after carrion deployment | 4.8891 | 37.668 | <0.001 | 0.00078 |
| Mg | Initial concentrations | -0.23351 | 43.278 | 0.8165 | 0.98614286 |
| Mg | Carrion plot after carrion deployment | -0.28 | 38.42 | 0.781 | 0.98614286 |
| Mg | Control plot after carrion deployment | -0.57277 | 38.356 | 0.5701 | 0.98614286 |
| N | Initial concentrations | 0.092197 | 42.313 | 0.927 | 0.98614286 |
| N | Carrion plot after carrion deployment | -0.033447 | 35.931 | 0.9735 | 0.98614286 |
| N | Control plot after carrion deployment | 2.0067 | 41.974 | 0.05125 | 0.38064 |
| Na | Initial concentrations | -0.033544 | 43.261 | 0.9734 | 0.98614286 |
| Na | Carrion plot after carrion deployment | -2.8412 | 21.816 | 0.009552 | 0.124176 |
| Na | Control plot after carrion deployment | 0.57005 | 45.979 | 0.5714 | 0.98614286 |
| P | Initial concentrations | 0.063294 | 39.503 | 0.9499 | 0.98614286 |
| P | Carrion plot after carrion deployment | -0.38964 | 40.967 | 0.6988 | 0.98614286 |
| P | Control plot after carrion deployment | 1.3071 | 41.92 | 0.1983 | 0.652925 |
| S | Initial concentrations | 0.20388 | 43.522 | 0.8394 | 0.98614286 |
| S | Carrion plot after carrion deployment | 0.45003 | 34.556 | 0.6555 | 0.98614286 |
| S | Control plot after carrion deployment | 0.94926 | 36.2 | 0.3488 | 0.8502 |

Table S2 All test statistics belonging to the macro elements; elemental concentrations related to estimated decomposition time. DT = estimated decomposition time; ST = sample type (carrion or control plot); DD = difference between estimated decomposition time and sampling date; INTER = interaction DT and ST.

| **Element** | **Variable** | **Sum of squares** | **Mean of squares** | **NumDF** | **DenDF** | **F value** | **p value** |
| --- | --- | --- | --- | --- | --- | --- | --- |
| C | DT | 1.3127E+16 | 1.3127E+16 | 1 | 1090031509 | 0.43742622 | 0.50836733 |
| C | ST | 3.5407E+16 | 3.5407E+16 | 1 | 7.0147E+20 | 1.17984996 | 0.27738654 |
| C | DD | 9.2689E+16 | 9.2689E+16 | 1 | 1090031218 | 3.08863666 | 0.07884084 |
| C | INTER | 3.7714E+16 | 3.7714E+16 | 1 | 5.5913E+20 | 1.2567298 | 0.262271 |
| Ca | DT | 2.9908E+12 | 2.9908E+12 | 1 | 10.0000041 | 0.73199166 | 0.41226848 |
| Ca | ST | 6.8586E+12 | 6.8586E+12 | 1 | 1.5367E+25 | 1.67862807 | 0.19510685 |
| Ca | DD | 1.9817E+12 | 1.9817E+12 | 1 | 10.0000041 | 0.48500978 | 0.50202802 |
| Ca | INTER | 5.1161E+12 | 5.1161E+12 | 1 | 1.3159E+25 | 1.25214546 | 0.2631431 |
| Fe | DT | 14583640.9 | 14583640.9 | 1 | 12.7243054 | 0.04981461 | 0.82692923 |
| Fe | ST | 605853609 | 605853609 | 1 | 2.1350388 | 2.069467 | 0.27943317 |
| Fe | DD | 347461556 | 347461556 | 1 | 17.6841507 | 1.18685473 | 0.29058857 |
| Fe | INTER | 666589480 | 666589480 | 1 | 19.9271185 | 2.27692781 | 0.14700466 |
| K | DT | 1.4733E+11 | 1.4733E+11 | 1 | 2.1108E+40 | 0.1044774 | 0.74652134 |
| K | ST | 9.7212E+10 | 9.7212E+10 | 1 | 2.1108E+40 | 0.06893693 | 0.79289095 |
| K | DD | 4.3384E+12 | 4.3384E+12 | 1 | 2.1108E+40 | 3.07650443 | 0.07943107 |
| K | INTER | 4.2886E+12 | 4.2886E+12 | 1 | 2.1108E+40 | 3.04119391 | 0.08117619 |
| Mg | DT | 9.1936E+11 | 9.1936E+11 | 1 | 9.99999973 | 0.45533135 | 0.51511126 |
| Mg | ST | 1.8268E+10 | 1.8268E+10 | 1 | 164716636 | 0.00904742 | 0.92422116 |
| Mg | DD | 2.7014E+11 | 2.7014E+11 | 1 | 9.99999982 | 0.13379039 | 0.72215288 |
| Mg | INTER | 670728247 | 670728247 | 1 | 2024478160 | 0.00033219 | 0.98545847 |
| N | DT | 9.2191E+12 | 9.2191E+12 | 1 | 13.5699768 | 0.35968834 | 0.55856134 |
| N | ST | 1.5831E+13 | 1.5831E+13 | 1 | 1.74190256 | 0.61766963 | 0.52457283 |
| N | DD | 3.0725E+13 | 3.0725E+13 | 1 | 12.4994108 | 1.19876534 | 0.29421807 |
| N | INTER | 1.921E+14 | 1.921E+14 | 1 | 218.760227 | 7.49500266 | 0.00669625 |
| Na | DT | 6.472E+10 | 6.472E+10 | 1 | 2.258E+35 | 0.28446085 | 0.59379221 |
| Na | ST | 7.9631E+10 | 7.9631E+10 | 1 | 2.258E+35 | 0.34999927 | 0.55411354 |
| Na | DD | 1.1143E+12 | 1.1143E+12 | 1 | 2.258E+35 | 4.89784011 | 0.02689031 |
| Na | INTER | 1.6855E+12 | 1.6855E+12 | 1 | 2.258E+35 | 7.40817162 | 0.00649283 |
| P | DT | 3.5616E+11 | 3.5616E+11 | 1 | 1.4541E+37 | 1.90163826 | 0.16789506 |
| P | ST | 1.8984E+11 | 1.8984E+11 | 1 | 1.4541E+37 | 1.01361189 | 0.31403909 |
| P | DD | 1.1777E+12 | 1.1777E+12 | 1 | 1.4541E+37 | 6.28786951 | 0.01215671 |
| P | INTER | 1127458546 | 1127458546 | 1 | 1.4541E+37 | 0.00601983 | 0.93815613 |
| S | DT | 921282136 | 921282136 | 1 | 14.1322454 | 0.00081794 | 0.97758389 |
| S | ST | 5.6203E+11 | 5.6203E+11 | 1 | 1.75357219 | 0.49898158 | 0.56192345 |
| S | DD | 1.1472E+12 | 1.1472E+12 | 1 | 12.4806654 | 1.01853362 | 0.3320336 |
| S | INTER | 4.7818E+12 | 4.7818E+12 | 1 | 287.310276 | 4.24538783 | 0.04025726 |

Table S3 All test statistics belonging to the trace elements; comparison carrion and control plot before and after carrion deployment (Welch Two Sample t-test). The column “Adjusted p” reports the p values after correction for multiple testing based on Benjamini & Hochberg (1995).

| **Element** | **Comparison** | **t-value** | **DF** | **p value** | **Ajusted p** |
| --- | --- | --- | --- | --- | --- |
| B | Initial concentrations | -0.55547 | 43.08 | 0.5814 | 0.98614286 |
| B | Carrion plot after carrion deployment | -0.067178 | 36.431 | 0.9468 | 0.98614286 |
| B | Control plot after carrion deployment | 2.9594 | 41.196 | 0.005091 | 0.0992745 |
| Ba | Initial concentrations | 0.25992 | 39.069 | 0.7963 | 0.98614286 |
| Ba | Carrion plot after carrion deployment | 1.3063 | 31.608 | 0.2009 | 0.652925 |
| Ba | Control plot after carrion deployment | 0.9692 | 45.964 | 0.3375 | 0.84919355 |
| Co | Initial concentrations | -0.21314 | 40.928 | 0.8323 | 0.98614286 |
| Co | Carrion plot after carrion deployment | -1.1188 | 22.747 | 0.2749 | 0.79415556 |
| Co | Control plot after carrion deployment | -0.58702 | 45.237 | 0.5601 | 0.98614286 |
| Cr | Initial concentrations | -0.12786 | 42.58 | 0.8989 | 0.98614286 |
| Cr | Carrion plot after carrion deployment | -1.9572 | 34.103 | 0.05856 | 0.38064 |
| Cr | Control plot after carrion deployment | -1.5426 | 33.427 | 0.1323 | 0.60702353 |
| Cu | Initial concentrations | 0.39921 | 41.905 | 0.6918 | 0.98614286 |
| Cu | Carrion plot after carrion deployment | -0.68156 | 24.307 | 0.502 | 0.98614286 |
| Cu | Control plot after carrion deployment | 0.32026 | 43.353 | 0.7503 | 0.98614286 |
| Mn | Initial concentrations | -0.24456 | 41.856 | 0.808 | 0.98614286 |
| Mn | Carrion plot after carrion deployment | -0.15662 | 38.803 | 0.8764 | 0.98614286 |
| Mn | Control plot after carrion deployment | 1.393 | 32.168 | 0.1732 | 0.652925 |
| Mo | Initial concentrations | 1.2521 | 23.792 | 0.2227 | 0.694824 |
| Mo | Carrion plot after carrion deployment | -0.82872 | 41.965 | 0.412 | 0.94517647 |
| Mo | Control plot after carrion deployment | -2.2103 | 45.61 | 0.03214 | 0.27854667 |
| Ni | Initial concentrations | 0.014969 | 43.307 | 0.9881 | 0.9881 |
| Ni | Carrion plot after carrion deployment | -1.5718 | 33.928 | 0.1253 | 0.60702353 |
| Ni | Control plot after carrion deployment | -1.3392 | 39.144 | 0.1882 | 0.652925 |
| Se | Initial concentrations | -0.42725 | 41.987 | 0.6714 | 0.98614286 |
| Se | Carrion plot after carrion deployment | -2.7918 | 27.276 | 0.009461 | 0.124176 |
| Se | Control plot after carrion deployment | -1.5784 | 24.052 | 0.1275 | 0.60702353 |
| Si | Initial concentrations | 0.33205 | 42.394 | 0.7415 | 0.98614286 |
| Si | Carrion plot after carrion deployment | -0.40962 | 40.959 | 0.6842 | 0.98614286 |
| Si | Control plot after carrion deployment | -0.69716 | 45.337 | 0.4893 | 0.98614286 |
| Sr | Initial concentrations | 0.17495 | 43.681 | 0.8619 | 0.98614286 |
| Sr | Carrion plot after carrion deployment | 0.1722 | 41.475 | 0.8641 | 0.98614286 |
| Sr | Control plot after carrion deployment | 0.4011 | 42.74 | 0.6903 | 0.98614286 |
| Zn | Initial concentrations | -0.50907 | 41.827 | 0.6134 | 0.98614286 |
| Zn | Carrion plot after carrion deployment | 0.13009 | 37.297 | 0.8972 | 0.98614286 |
| Zn | Control plot after carrion deployment | 2.5918 | 45.53 | 0.01279 | 0.14251714 |

Table S4 All test statistics belonging to the trace elements; elemental concentrations related to estimated decomposition time. DT = estimated decomposition time; ST = sample type (carrion or control plot); DD = difference between estimated decomposition time and sampling date; INTER = interaction DT and ST.

| **Element** | **Variable** | **Sum of squares** | **Mean of squares** | **NumDF** | **DenDF** | **F value** | **p value** |
| --- | --- | --- | --- | --- | --- | --- | --- |
| B | DT | 7.02938544 | 7.02938544 | 1 | 10 | 1.05131055 | 0.3293686 |
| B | ST | 15.5163452 | 15.5163452 | 1 | 11 | 2.32061503 | 0.15588777 |
| B | DD | 0.49870255 | 0.49870255 | 1 | 10 | 0.07458565 | 0.79033058 |
| B | INTER | 41.5118613 | 41.5118613 | 1 | 11 | 6.20848837 | 0.02994568 |
| Ba | DT | 1792.70244 | 1792.70244 | 1 | 21 | 0.81510159 | 0.37685602 |
| Ba | ST | 187.817068 | 187.817068 | 1 | 21 | 0.08539621 | 0.77298118 |
| Ba | DD | 3162.84433 | 3162.84433 | 1 | 21 | 1.43807438 | 0.24380787 |
| Ba | INTER | 0.67358687 | 0.67358687 | 1 | 21 | 0.00030626 | 0.9862026 |
| Co | DT | 49.6723948 | 49.6723948 | 1 | 9.33976809 | 0.09045619 | 0.77018594 |
| Co | ST | 772.284216 | 772.284216 | 1 | 2.08251623 | 1.40637251 | 0.35332848 |
| Co | DD | 676.105377 | 676.105377 | 1 | 15.2707586 | 1.2312255 | 0.28433737 |
| Co | INTER | 648.541044 | 648.541044 | 1 | 18.5788111 | 1.18102932 | 0.29104562 |
| Cr | DT | 78.6599629 | 78.6599629 | 1 | 9.16070146 | 0.10117922 | 0.75755092 |
| Cr | ST | 345.519966 | 345.519966 | 1 | 2.02947236 | 0.44443754 | 0.57273019 |
| Cr | DD | 214.406186 | 214.406186 | 1 | 10.2654169 | 0.2757877 | 0.61062963 |
| Cr | INTER | 128.669622 | 128.669622 | 1 | 11.7347467 | 0.16550595 | 0.69145918 |
| Cu | DT | 16.0976 | 16.0976 | 1 | 6.07239131 | 3.14906525 | 0.12573503 |
| Cu | ST | 0.18020686 | 0.18020686 | 1 | 1.20431919 | 0.03525266 | 0.8776779 |
| Cu | DD | 0.13906537 | 0.13906537 | 1 | 7.80618058 | 0.02720442 | 0.87318403 |
| Cu | INTER | 15.0485882 | 15.0485882 | 1 | 9.8846527 | 2.94385413 | 0.11732183 |
| Mn | DT | 81530.0651 | 81530.0651 | 1 | 13.5802098 | 0.02942084 | 0.86634054 |
| Mn | ST | 3581134.82 | 3581134.82 | 1 | 2.1447613 | 1.29228393 | 0.36664857 |
| Mn | DD | 2732047.12 | 2732047.12 | 1 | 18.1917558 | 0.98588318 | 0.33377492 |
| Mn | INTER | 3585375.06 | 3585375.06 | 1 | 20.1694149 | 1.29381406 | 0.26867906 |
| Mo | DT | 0.01567845 | 0.01567845 | 1 | 12.1520233 | 0.03364118 | 0.85749694 |
| Mo | ST | 0.84438901 | 0.84438901 | 1 | 1.3042557 | 1.81180144 | 0.36565975 |
| Mo | DD | 0.00790445 | 0.00790445 | 1 | 11.6791441 | 0.01696053 | 0.89859855 |
| Mo | INTER | 0.50236704 | 0.50236704 | 1 | 10.6979652 | 1.07792655 | 0.3220592 |
| Ni | DT | 36.7937051 | 36.7937051 | 1 | 21 | 0.40678927 | 0.53049734 |
| Ni | ST | 6.56893048 | 6.56893048 | 1 | 21 | 0.07262575 | 0.79018164 |
| Ni | DD | 29.9049865 | 29.9049865 | 1 | 21 | 0.33062796 | 0.57139934 |
| Ni | INTER | 11.2570259 | 11.2570259 | 1 | 21 | 0.12445709 | 0.72776611 |
| Se | DT | 0.40495734 | 0.40495734 | 1 | 20.737795 | 0.17035349 | 0.68403388 |
| Se | ST | 9.82594507 | 9.82594507 | 1 | 1.85863846 | 4.13348242 | 0.18867785 |
| Se | DD | 1.01306804 | 1.01306804 | 1 | 20.9955552 | 0.42616755 | 0.52096024 |
| Se | INTER | 24.3844513 | 24.3844513 | 1 | 20.7725288 | 10.2578124 | 0.00431749 |
| Si | DT | 9579503313 | 9579503313 | 1 | 12.054678 | 0.05295438 | 0.82185862 |
| Si | ST | 4.3358E+11 | 4.3358E+11 | 1 | 1.71290695 | 2.39675634 | 0.28135111 |
| Si | DD | 6.161E+10 | 6.161E+10 | 1 | 11.5468996 | 0.34057119 | 0.57072329 |
| Si | INTER | 9.481E+11 | 9.481E+11 | 1 | 105.988908 | 5.24101409 | 0.02404235 |

Table S4 *Continued*.

| **Element** | **Variable** | **Sum of squares** | **Mean of squares** | **NumDF** | **DenDF** | **F value** | **p value** |
| --- | --- | --- | --- | --- | --- | --- | --- |
| Sr | DT | 375.480893 | 375.480893 | 1 | 9.99999935 | 0.88873724 | 0.36803724 |
| Sr | ST | 362.304809 | 362.304809 | 1 | 11.0000003 | 0.85755036 | 0.37429574 |
| Sr | DD | 439.607382 | 439.607382 | 1 | 9.99999935 | 1.04052019 | 0.33174408 |
| Sr | INTER | 577.57412 | 577.57412 | 1 | 11.0000003 | 1.36707789 | 0.26702725 |
| Zn | DT | 2313.82412 | 2313.82412 | 1 | 21 | 3.62575808 | 0.07068372 |
| Zn | ST | 154.516527 | 154.516527 | 1 | 21 | 0.24212711 | 0.62777858 |
| Zn | DD | 15.6651372 | 15.6651372 | 1 | 21 | 0.02454724 | 0.87699702 |
| Zn | INTER | 1938.26155 | 1938.26155 | 1 | 21 | 3.03725223 | 0.09599996 |

Table S5 All test statistics belonging to the ecotoxic elements; comparison carrion and control plot before and after carrion deployment (Welch Two Sample t-test). The column “Adjusted p” reports the p values after correction for multiple testing based on Benjamini & Hochberg (1995).

| **Element** | **Comparison** | **t-value** | **DF** | **p value** | **Adjusted p** |
| --- | --- | --- | --- | --- | --- |
| Al | Initial concentrations | -0.26548 | 42.153 | 0.7919 | 0.98614286 |
| Al | Carrion plot after carrion deployment | -1.4283 | 33.939 | 0.1623 | 0.652925 |
| Al | Control plot after carrion deployment | -1.6003 | 42.026 | 0.117 | 0.60702353 |
| As | Initial concentrations | 0.71657 | 35.462 | 0.4783 | 0.98614286 |
| As | Carrion plot after carrion deployment | -1.4227 | 37.053 | 0.1632 | 0.652925 |
| As | Control plot after carrion deployment | -1.9525 | 38.602 | 0.05816 | 0.38064 |
| Cd | Initial concentrations | -0.53795 | 43.177 | 0.5934 | 0.98614286 |
| Cd | Carrion plot after carrion deployment | 0.5639 | 40.943 | 0.5759 | 0.98614286 |
| Cd | Control plot after carrion deployment | 1.0773 | 42.025 | 0.2875 | 0.80089286 |
| Pb | Initial concentrations | -0.27992 | 43.559 | 0.7809 | 0.98614286 |
| Pb | Carrion plot after carrion deployment | -1.0492 | 39.66 | 0.3004 | 0.80797241 |
| Pb | Control plot after carrion deployment | -1.3679 | 44.815 | 0.1782 | 0.652925 |

Table S6 All test statistics belonging to the ecotoxic elements; elemental concentrations related to estimated decomposition time. DT = estimated decomposition time; ST = sample type (carrion or control plot); DD = difference between estimated decomposition time and sampling date; INTER = interaction DT and ST.

| **Element** | **Variable** | **Sum of squares** | **Mean of squares** | **NumDF** | **DenDF** | **F value** | **p value** |
| --- | --- | --- | --- | --- | --- | --- | --- |
| Al | DT | 9.4707E+11 | 9.4707E+11 | 1 | 59.9426113 | 0.04590832 | 0.83106974 |
| Al | ST | 3.6618E+13 | 3.6618E+13 | 1 | 2.18048249 | 1.77499799 | 0.30491089 |
| Al | DD | 1.4279E+13 | 1.4279E+13 | 1 | 127.224176 | 0.6921496 | 0.40699304 |
| Al | INTER | 9.0523E+13 | 9.0523E+13 | 1 | 252.158141 | 4.38800143 | 0.03719078 |
| As | DT | 0.04128477 | 0.04128477 | 1 | 20.4015076 | 0.0163626 | 0.89946765 |
| As | ST | 2.28946789 | 2.28946789 | 1 | 1.76924779 | 0.90739634 | 0.45235084 |
| As | DD | 1.23338606 | 1.23338606 | 1 | 20.059729 | 0.48883411 | 0.49247588 |
| As | INTER | 5.90469577 | 5.90469577 | 1 | 19.7213454 | 2.34023781 | 0.14195295 |
| Cd | DT | 0.13606091 | 0.13606091 | 1 | 21 | 3.54006562 | 0.0738392 |
| Cd | ST | 0.03605708 | 0.03605708 | 1 | 21 | 0.9381419 | 0.34378 |
| Cd | DD | 0.32261651 | 0.32261651 | 1 | 21 | 8.39391417 | 0.0086187 |
| Cd | INTER | 0.05262717 | 0.05262717 | 1 | 21 | 1.36926643 | 0.25504705 |
| Pb | DT | 137.289558 | 137.289558 | 1 | 9.99999013 | 1.07791148 | 0.32361765 |
| Pb | ST | 25.9516058 | 25.9516058 | 1 | 10.999986 | 0.20375573 | 0.66047585 |
| Pb | DD | 1.1650266 | 1.1650266 | 1 | 9.99999014 | 0.00914706 | 0.92569575 |
| Pb | INTER | 16.9246475 | 16.9246475 | 1 | 10.999986 | 0.13288171 | 0.72237357 |

Table S7 All test statistics belonging to the soil pH; comparison carrion and control plot before and after carrion deployment (Welch Two Sample t-test). The column “Adjusted p” reports the p values after correction for multiple testing based on Benjamini & Hochberg (1995).

| **Comparison** | **t-value** | **DF** | **p value** | **Adjusted p** |
| --- | --- | --- | --- | --- |
| Initial concentrations | -0.9913 | 43.523 | 0.327 | 0.84919355 |
| Carrion plot after carrion deployment | -3.7801 | 33.954 | 0.0006061 | 0.0157586 |
| Control plot after carrion deployment | 0.40606 | 45.726 | 0.6866 | 0.98614286 |

Table S8 All test statistics belonging to the soil pH; elemental concentrations related to estimated decomposition time. DT = estimated decomposition time; ST = sample type (carrion or control plot); DD = difference between estimated decomposition time and sampling date; INTER = interaction DT and ST.

| **Variable** | **Sum of squares** | **Mean of squares** | **NumDF** | **DenDF** | **F value** | **p value** |
| --- | --- | --- | --- | --- | --- | --- |
| DT | 0.08069241 | 0.08069241 | 1 | 5.05199256 | 0.5063851 | 0.50820897 |
| ST | 0.19948388 | 0.19948388 | 1 | 1.93523841 | 1.25186081 | 0.38283651 |
| DD | 0.1305933 | 0.1305933 | 1 | 10.8574124 | 0.81953809 | 0.38496384 |
| INTER | 0.03043577 | 0.03043577 | 1 | 15.322299 | 0.19099963 | 0.66818259 |


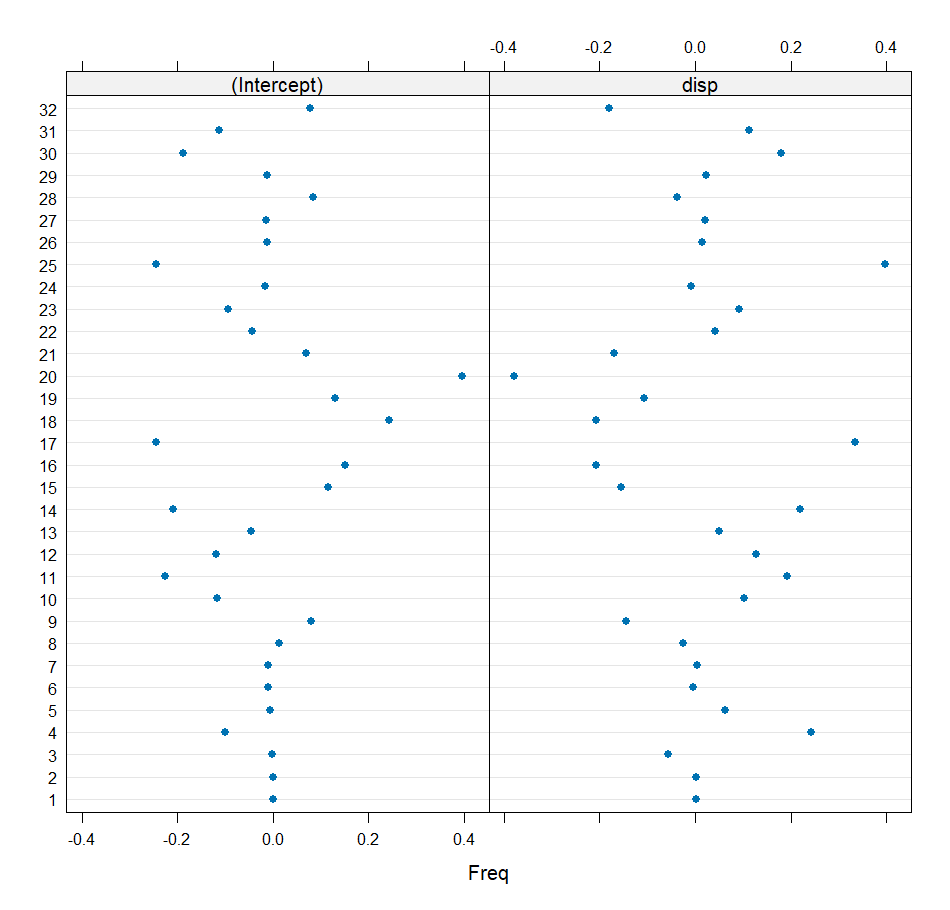


Figure S1 Test for leverage based on Cook's distance in the linear mixed-effects models (Table S2+4+6+8).
